# Supplementary material for: How effective are UK-based support interventions and services targeted at adults who have experienced domestic and sexual violence and abuse at improving their safety and wellbeing? A systematic review protocol
Source: PLoS One. 2023 Dec 7;18(12):e0289192. doi: 10.1371/journal.pone.0289192 (PMC10703258; doi:10.1371/journal.pone.0289192)
Supplement: S1 File — (PDF) [file pone.0289192.s001.pdf]

S1 Glossary

Advocacy: Adapted from the National Institute for Health and Clinical Excellence (NICE) Domestic Violence and Abuse: Multi-agency Working public health guideline (PH50). In general, advocacy for people who have experienced DSVA includes: legal, housing and financial advice: access to and use of community resources such as refuges, emergency housing and psychological interventions; and safety planning advice. The activities may differ according to the level of risk facing the person. Crisis advocacy involves working with the person for a limited period of time (they may then be referred on to more specialist agencies). Practitioners providing advocacy can also provide ongoing support and informal counselling. The intensity of the advocacy provided may vary. It may last for a year – or longer, if the person is particularly vulnerable.

Domestic and sexual violence and abuse (DSVA): This review works to the UK government definition of domestic violence and abuse, and The Istanbul Convention, The World Health Organisation, and The Rome Statute of the International Criminal Court's (ICC) Elements of Crimes definitions of sexual violence and abuse. In brief, domestic violence and abuse is defined as behaviour within an intimate relationship or family which includes acts of physical violence, sexual violence, emotional or psychological abuse, and controlling behaviours. This also includes 'honour'-based violence and FGM. Sexual violence and abuse are defined as any non-consensual act of sexual nature, extending to attempts to obtain a sexual act, unwanted sexual comments or advances, or acts to traffic, using force, threat of force or coercion.

Floating support: Adapted from the NICE PH50 guideline. Floating support is a housing service designed to prevent tenancy breakdowns. Floating support can also provide help with: keeping safe and security measures; accessing legal advice and options; welfare benefits; budgeting and debts; life skills; resettlement or re-housing; accessing community services; form filling; pre-tenancy support; training, education and employment.

Independent domestic violence advisers (IDVAs): Adapted from the NICE PH50 guideline. Also known as independent domestic violence advocates, IDVAs work primarily with people at high risk of domestic violence and abuse, independently of any one agency, to secure their safety and the safety of their children. Serving as the primary point of contact, IDVAs normally work with their clients from the point of crisis to assess the level of risk, discuss the options and develop plans that address their immediate safety, as well as longer-term solutions. In many areas they are funded by the local community safety partnership, in some areas they are funded by the police or local authorities.

Intervention: Adapted from the NICE Glossary. A treatment, service, project, or programme.

Intimate partner violence (IPV): Adapted from the World Health Organisation's definition. Intimate partner violence refers to behaviour within an intimate relationship that causes physical, sexual or psychological harm, including acts of physical aggression, sexual coercion, psychological abuse and controlling behaviours. This definition covers violence by both current and former spouses and partners.

Organisation: In the context of this review, organisations refer exclusively to those that carry out activities relating to DSVA. Organisations may include both specialist service providers, and organisations that do not directly provider services (such as women's rights organisations that may focus on activities such as campaigning, research, supporting specialist services, training, and education, for issues relating to DSVA, for example).

People who have experienced DSVA: Throughout this review, people who experience DSVA refers to those who are victims or survivors of DSVA.

Primary, secondary, and tertiary prevention: Primary prevention refers to any action, strategy or policy that works to stop domestic violence from occurring in the first place. Primary prevention seeks to reduce the overall likelihood that anyone will become a victim or perpetrator of domestic violence by creating conditions that make violence less likely to occur. Secondary prevention refers to intervening and responding to violence that has

already occurred in order to stop violence from happening again. Secondary prevention interventions and services can include refuge, counselling, and safety planning. Tertiary prevention refers to ongoing support to victims and ongoing accountability for abusers. Tertiary prevention interventions and services address the long-term consequences of domestic violence. They can include support groups and other resources for survivors to help them heal so that they do not experience abuse again. They can also include perpetrator programs for abusers to prevent them from continuing to perpetrate violence in the future.

Refuge or shelter: Adapted from the NICE PH50 guideline. Residential service – a safe house – provided for adults (usually women) and children who are experiencing domestic violence and abuse.

Safety planning: Adapted from the NICE PH50 guideline. An intervention to help people judge their risk of violence, identify the warning signs and develop plans on what to do when violence is imminent or is happening.

Service provider: Primary, community, including third sector, secondary and tertiary care providers of health and social care services, including prison health services and criminal justice agencies. In the context of DSVA, the service providers are predominantly third sector providers. Third sector service providers may be specialist DSVA providers (i.e., they only provide services relating to DSVA) or non-specialist-DSVA providers (i.e., they provide specialist support services for DSVA as well as support services for other issues). Outside of the third sector, service providers such as the NHS, police and courts may provide some DSVA specific services.

Third sector organisations: In this review the definition of third sector services provided by the National Audit Office has been adopted. 'Third sector organisations' is a term used to describe the range of organisations that are neither public sector nor private sector. It includes voluntary and community organisations (both registered charities and other

organisations such as associations, self-help groups and community groups), social enterprises, mutuals and co-operatives. Third sector organisations generally: are independent of government. This is also an important part of the history and culture of the sector; are ‘value-driven’. This means they are motivated by the desire to achieve social goals (for example, improving public welfare, the environment or economic well-being) rather than the desire to distribute profit; and reinvest any surpluses generated in the pursuit of their goals. For this reason they are sometimes called ‘not-for-profit organisations’. A better term is ‘not-for-personal-profit’. In many cases, third sector organisations need to make surpluses (or ‘profits’) to be financially sustainable.
