# Supplementary material for: How effective are UK-based support interventions and services targeted at adults who have experienced domestic and sexual violence and abuse at improving their safety and wellbeing? A systematic review protocol
Source: PLoS One. 2023 Dec 7;18(12):e0289192. doi: 10.1371/journal.pone.0289192 (PMC10703258; doi:10.1371/journal.pone.0289192)
Supplement: S2 File — (PDF) [file pone.0289192.s002.pdf]

S3 Example preliminary search strategy

1. exp United kingdom/
2. (national health service* or nhs*).ti,ab,in.
3. (english not ((published or publication* or translat* or written or language* or speak* or literature or citation*) adj5 english)).ti,ab.
4. (gb or "g.b." or britain* or (british* not "british columbia") or uk or "u.k." or united kingdom* or (england* not "new england") or northern ireland* or northern irish* or scotland* or scottish* or ((wales or "south wales") not "new south wales") or welsh*).ti,ab,jw,in.
5. (bangor or "bangor's" or cardiff or "cardiff's" or newport or "newport's" or st asaph or "st asaph's" or st davids or swansea or "swansea's").ti,ab,in,cq.
6. (bath or "bath's" or ((birmingham not alabama*) or ("birmingham's" not alabama*) or bradford or "bradford's" or brighton or "brighton's" or bristol or "bristol's" or carlisle* or "carlisle's" or (cambridge not (massachusetts* or boston* or harvard*)) or ("cambridge's" not (massachusetts* or boston* or harvard*)) or (canterbury not zealand*) or ("canterbury's" not zealand*) or chelmsford or "chelmsford's" or chester or "chester's" or chichester or "chichester's" or coventry or "coventry's" or derby or "derby's" or (durham not (carolina* or nc)) or ("durham's" not (carolina* or nc)) or ely or "ely's" or exeter or "exeter's" or gloucester or "gloucester's" or hereford or "hereford's" or hull or "hull's" or lancaster or "lancaster's" or leeds* or leicester or "leicester's" or (lincoln not nebraska*) or ("lincoln's" not nebraska*) or (liverpool not (new south wales* or nsw)) or ("liverpool's" not (new south wales* or nsw)) or ((london not (ontario* or ont or toronto*)) or ("london's" not (ontario* or ont or toronto*)) or manchester or "manchester's" or (newcastle not (new south wales* or nsw)) or ("newcastle's" not (new south wales* or nsw)) or norwich or "norwich's" or nottingham or "nottingham's" or oxford or "oxford's" or peterborough or "peterborough's" or plymouth or "plymouth's" or portsmouth or "portsmouth's" or preston or "preston's" or ripon or "ripon's" or salford or "salford's" or salisbury or "salisbury's" or sheffield or "sheffield's" or southampton or "southampton's" or st albans or stoke or "stoke's" or sunderland or "sunderland's" or truro or "truro's" or wakefield or "wakefield's" or wells or westminster or "westminster's" or winchester or "winchester's" or wolverhampton or "wolverhampton's" or (worcester not (massachusetts* or boston* or harvard*)) or ("worcester's" not (massachusetts* or boston* or harvard*)) or (york not ("new york*" or ny or ontario* or ont or toronto*)) or ("york's" not ("new york*" or ny or ontario* or ont or toronto*))))).ti,ab,in.
7. (aberdeen or "aberdeen's" or dundee or "dundee's" or edinburgh or "edinburgh's" or glasgow or "glasgow's" or inverness or (perth not australia*) or ("perth's" not australia*) or stirling or "stirling's").ti,ab,in.
8. (armagh or "armagh's" or belfast or "belfast's" or lisburn or "lisburn's" or londonderry or "londonderry's" or derry or "derry's" or newry or "newry's").ti,ab,in.
9. ("City of London" or Bedfordshire or Berkshire or Bristol or Buckinghamshire or Cambridgeshire or Cheshire or Cornwall or Cumbria or Derbyshire or Devon or Dorset or Durham or Yorkshire or Essex or Gloucestershire or "greater london" or "greater manchester" or "isle of wight" or "west midlands" or sussex or Hampshire or Herefordshire or Hertfordshire or Kent or Lancashire Leicestershire or Lincolnshire or Merseyside or Norfolk or Northamptonshire or Northumberland or Nottinghamshire or Oxfordshire or Rutland or Shropshire or Somerset or Staffordshire or Suffolk or Surrey or "Tyne and Wear" or Warwickshire or Wiltshire or Worcestershire or Aberdeenshire or Angus or Forfarshire or Argyll* or Ayrshire or "County of Ayr" or "County of Banff" or "County of Berwick" or "County of Bute" or "County of Clackmannan" or "East Lothian" or "Ross and Cromarty" or "West Lothian" or Banffshire or Berwickshire or Buteshire or Caithness or Clackmannanshire or Dumfriesshire or Dunbartonshire or Fife or Invernessshire or Kincardineshire or Kinrossshire or Kirkcudbrightshire or Lanarkshire or Midlothian or Moray or Nairnshire or Orkney or Peebleshire or Perthshire or Renfrewshire or Roxburghshire or Selkirkshire or Shetland or Stirlingshire or Sutherland or Wigtownshire or "Blaenau Gwent" or "Isle of Anglesey" or "Merthyr Tydfil" or "Neath Port Talbot" or "Rhondda Cynon Taff" or "Vale of Glamorgan" or Bridgend or Caerphilly or Cardiff or Carmarthenshire or Ceredigion or Conwy or Denbighshire or Flintshire or Gwynedd or Wrexham or Antrim or Armagh or Down or Fermanagh or Londonderry or derry or Swansea or Torfaen or Newport or Pembrokeshire or Powys or Monmouthshire or Tyrone).ti,ab,in.
10. (exp africa/ or exp americas/ or exp antarctic regions/ or exp arctic regions/ or exp asia/ or exp australia/ or exp oceania/) not (exp United Kingdom/ or europe/)
11. 1 or 2 or 3 or 4 or 5 or 6 or 7 or 8 or 9
12. 11 not 10

13. (violen* adj3 (sexual or domestic or child or partner* or spous* or couple* or family or interpersonal or date or dating or gender based or coercive or conjugal or victim* or survivor* or perpetrat* or technology-facilitated or wom#n or image based or honor based or obstetric)).ti,ab,kw.
14. (abus* adj3 (sexual or domestic or child or partner* or spous* or family or couple* or gender based or wom#n or wife or wives or victim* or survivor* or perpetrat* or financ* or economic or image-based or technology-facilitated)).ti,ab,kw.
15. (assault* adj3 (sexual or partner* or spous* or victim* or survivor* or perpetrat* or couple*)).ti,ab,kw.
16. (child adj3 maltreat*).ti,ab,kw.
17. (sexual adj3 harass*).ti,ab,kw.
18. (batter* adj3 (wom#n or wife or wives)).ti,ab,kw.
19. rape*.ti,ab,kw.
20. (beat* adj3 (partner* or spous* or wom#n or wife or wives or m#n or husband*)).ti,ab,kw.
21. (sexual adj3 coercion).ti,ab,kw.
22. female genital mutilation.ti,ab,kw.
23. forced marriage.ti,ab,kw.
24. sex traffic*.ti,ab,kw.
25. revenge porn.ti,ab,kw.
26. controlling behaviour.ti,ab,kw.
27. prostitut*.ti,ab,kw.
28. sex work*.ti,ab,kw.
29. stalking.ti,ab,kw.
30. (drink adj3 spik*).ti,ab,kw.
31. exp battered woman/
32. exp domestic violence/ or exp child abuse/ or exp spouse abuse/ or exp gender-based violence/ or exp intimate partner violence/ or exp physical abuse/ or exp rape/
33. exp partner violence/
34. exp family violence/
35. exp coercion/
36. exp emotional abuse/
37. exp sexual violence/ or exp sexual abuse/ or exp sexual assault/
38. 16 or 17 or 18 or 19 or 20 or 21 or 22 or 23 or 24 or 25 or 26 or 27 or 28 or 29 or 30 or 31 or 32 or 33 or 34 or 35 or 36 or 37
39. domestic abuse service*.ti,ab,kw.
40. women's project*.ti,ab,kw.
41. women's resource centre.ti,ab,kw.
42. safety unit*.ti,ab,kw.
43. domestic abuse support service*.ti,ab,kw.
44. domestic abuse team*.ti,ab,kw.
45. domestic violence intervention*.ti,ab,kw.
46. women's service*.ti,ab,kw.
47. women's rights organi#ation*.ti,ab,kw.
48. domestic abuse advice and support.ti,ab,kw.
49. women's centre*.ti,ab,kw.
50. domestic abuse project*.ti,ab,kw.
51. domestic and sexual abuse project*.ti,ab,kw.
52. victim care service*.ti,ab,kw.
53. support centre*.ti,ab,kw.
54. women's welfare.ti,ab,kw.
55. speciali* service*.ti,ab,kw.
56. speciali* sexual violence service*.ti,ab,kw.
57. speciali* domestic violence service*.ti,ab,kw.
58. speciali* support.ti,ab,kw.
59. speciali* violence and abuse service*.ti,ab,kw.
60. speciali* women's voluntary sector service*.ti,ab,kw.
61. service* for survivors.ti,ab,kw.
62. victim service*.ti,ab,kw.
63. victim support*.ti,ab,kw.
64. violence against women and girls service*.ti,ab,kw.
65. domestic violence support service*.ti,ab,kw.
66. community based service*.ti,ab,kw.
67. sexual assault referral centre*.ti,ab,kw.
68. rape crisis.ti,ab,kw.
69. third sector.ti,ab,kw.
70. second tier.ti,ab,kw.
71. crisis intervention.ti,ab,kw.
72. independent domestic violence advoca*.ti,ab,kw.
73. IDVA.ti,ab,kw.
74. independent sexual violence advoca*.ti,ab,kw.
75. ISVA.ti,ab,kw.
76. shelter*.ti,ab,kw.
77. refuge.ti,ab,kw.
78. refuges.ti,ab,kw.
79. advoca*.ti,ab,kw.
80. counsel?ing.ti,ab,kw.
81. outreach.ti,ab,kw.
82. helpline*.ti,ab,kw.
83. emergency shelter*.ti,ab,kw.
84. supportive hous*.ti,ab,kw.
85. violence against women service*.ti,ab,kw.
86. recovery service*.ti,ab,kw.
87. speciali* victim service*.ti,ab,kw.
88. independent domestic violence advisor*.ti,ab,kw.
89. independent sexual violence advisor*.ti,ab,kw.
90. resettlement.ti,ab,kw.
91. floating support.ti,ab,kw.
92. drop-in.ti,ab,kw.
93. online chat.ti,ab,kw.
94. support group*.ti,ab,kw.
95. violence advocacy project*.ti,ab,kw.
96. group work program*.ti,ab,kw.
97. information and advice.ti,ab,kw.
98. (dedicated adj4 service*).ti,ab,kw.
99. charit*.ti,ab,kw.
100. independent.ti,ab,kw.
101. voluntary.ti,ab,kw.
102. public sector.ti,ab,kw.
103. specialist.ti,ab,kw.
104. speciali?ed.ti,ab,kw.
105. non-governmental organi#ation*.ti,ab,kw.
106. nonprofit organi#ation*.ti,ab,kw.
107. not-for-profit organi#ation*.ti,ab,kw.
108. by and for service*.ti,ab,kw.
109. by and for organi#ation*.ti,ab,kw.
110. therapy.ti,ab,kw.
111. empowerment.ti,ab,kw.

112. case management.ti,ab,kw.
113. stress management.ti,ab,kw.
114. safety behaviour*.ti,ab,kw.
115. safety strateg*.ti,ab,kw.
116. legal advice.ti,ab,kw.
117. mindfulness based stress reduction.ti,ab,kw.
118. home visit*.ti,ab,kw.
119. sanctuar*.ti,ab,kw.
120. safe house*.ti,ab,kw.
121. flexible fund*.ti,ab,kw.
122. tenant support.ti,ab,kw.
123. perpetrator programme*.ti,ab,kw.
124. Peer support.ti,ab,kw.
125. Support service*.ti,ab,kw.
126. Independent domestic abuse advocate*.ti,ab,kw.
127. Independent sexual abuse advocate*.ti,ab,kw.
128. Support intervention*.ti,ab,kw.
129. Finacial advice.ti,ab,kw.
130. Befriend*.ti,ab,kw.
131. MARAC.ti,ab,kw.
132. Multi agency risk assessment conference*.ti,ab,kw.
133. Housing worker*.ti,ab,kw.
134. 39 or 40 or 41 or 42 or 43 or 44 or 45 or 46 or 47 or 48 or 49 or 50 or 51 or 52 or 53 or 54 or 55 or 56 or 57 or 58 or 59 or 60 or 61 or 62 or 63 or 64 or 65 or 66 or 67 or 68 or 69 or 70 or 71 or 72 or 73 or 74 or 75 or 76 or 77 or 78 or 79 or 80 or 81 or 82 or 83 or 84 or 85 or 86 or 87 or 88 or 89 or 90 or 91 or 92 or 93 or 94 or 95 or 96 or 97 or 98 or 99 or 100 or 101 or 102 or 103 or 104 or 105 or 106 or 107 or 108 or 109 or 110 or 111 or 112 or 113 or 114 or 115 or 116 or 117 or 118 or 119 or 120 or 121 or 122 or 123
135. 12 and 38 and 124
136. limit 125 to (english language and humans)
